# The prescribing pattern of sodium-glucose cotransporter-2 inhibitors and glucagon-like peptide-1 receptor agonists in patient with type two diabetes mellitus: A two-center retrospective cross-sectional study

**DOI:** 10.3389/fpubh.2022.1031306

**Published:** 2022-10-28

**Authors:** Ghazwa B. Korayem, Omar A. Alshaya, Albandari A. Alghamdi, Shahad S. Alanazi, Renad T. Almutib, Mahdi Alsaileek, Abdulrahman Alrashidi, Nasser Aldosari, Nader Bin Sheraim, Majed S. Al Yami, Omar A. Almohammed

**Affiliations:** ^1^Department of Pharmacy Practice, College of Pharmacy, Princess Nourah bint Abdulrahman University, Riyadh, Saudi Arabia; ^2^Department of Pharmacy Practice, College of Pharmacy, King Saud Bin Abdulaziz University for Health Sciences, Riyadh, Saudi Arabia; ^3^Pharmaceutical Care Department, King Abdulaziz Medical City, Riyadh, Saudi Arabia; ^4^King Abdullah International Medical Research Center, Riyadh, Saudi Arabia; ^5^Pharmaceutical Care Services, King Abdullah Bin Abdulaziz University Hospital, Riyadh, Saudi Arabia; ^6^Department of Clinical Pharmacy, College of Pharmacy, King Saud University, Riyadh, Saudi Arabia; ^7^Pharmacoeconomics Research Unit, College of Pharmacy, King Saud University, Riyadh, Saudi Arabia

**Keywords:** type 2 diabetes, underprescription, sodium-glucose cotransporter-2 inhibitors, glucagon-like peptide-1 receptor agonists, comorbidities

## Abstract

**Background:**

The use of sodium-glucose cotransporter-2 inhibitors (SGLT2i) and glucagon-like peptide-1 receptor agonists (GLP-1 RA) in patients with type 2 diabetes mellitus (T2DM) remains limited, especially in those with other compelling indications. Thus, this study aimed to describe the prescribing patterns of GLP-1-RA and SGLT2i in patients with T2DM and to determine the factors that affect the prescribing of these medications.

**Methods:**

This multicenter retrospective cross-sectional study reviewed the electronic health records of adult patients diagnosed with T2DM who received care between January and December 2020. The patients were classified according to their compelling indications into “patients who are more likely” to benefit from SGLT2i or GLP-1 RA and “patients who are less likely” to benefit from them. They were then further categorized depending on whether these medications were prescribed.

**Results:**

A total of 1,220 patients were included; most were female (56.9%). SGLT2i or GLP-1 RA were preferably prescribed in only 19% of the patients for reasons including BMI ≥ 27 kg/m^2^ (85.6%), uncontrolled T2DM (68.5%), high risk for ASCVD (23.9%), or established ASCVD (14%). The remaining 81.0% were underprescribed these agents. Patients at an older age or with a history of stroke or transient ischemic attack had higher odds of being underprescribed (OR 1.02; 95% CI: 1.01–1.03 and OR 2.86; 95% CI: 1.33–6.15), respectively.

**Conclusion:**

The results concur with those of previous studies highlighting the underutilization of GLP-1 RA and SGLT2i in patients with T2DM but also with compelling indications. To optimize the use of GLP-1 RA and SGLT2i for their additional benefits, prescribers need to assess the benefits of using these agents in patients who would likely benefit from them, regardless of DM control.

## Background

Sodium-glucose cotransporter-2 inhibitors (SGLT2i) and glucagon-like peptide-1 receptor agonists (GLP-1 RA) are among the emerging classes of antidiabetic medications for the management of type 2 diabetes mellitus (T2DM). Several trials have demonstrated that SGLT2i and GLP-1 RA can reduce major adverse cardiovascular events (MACE), death from cardiovascular diseases (CVD), and heart failure (HF) hospitalization, as well as delay the progression of chronic kidney disease (CKD) in patients at high risk of or with established atherosclerotic cardiovascular disease (ASCVD) (1–8). Thus, the treatment decision for patients with T2DM should follow a patient-centered approach based on the presence of CVD and renal comorbidities, the agents' efficacy and safety, and the patient's preference (9, 10).

Since SGLT2i and GLP-1 RA exhibit cardiovascular, renal, and weight-reduction benefits independent of their blood-glucose-lowering effect (10), recent diabetes guidelines recommend using these medication classes for T2DM patients with established ASCVD, HF, or CKD, or for those at high risk of ASCVD (11, 12). Moreover, the Kidney Disease: Improving Global Outcomes (KDIGO) Foundation and the American Diabetes Association (ADA) recommend first-line treatment with metformin or SGLT2i in patients with CKD and T2DM (13, 14). The updated American Heart Association and the European Society of Cardiology guidelines recommend using SGLT2i in patients with HF with or without T2DM (15, 16). In addition, due to the significant weight-reduction effect of SGLT2i and GLP-1 RA, these agents should be considered for individuals with T2DM who are overweight or obese (9, 17).

Despite the proven cardiorenal benefits and weight-reduction effects of SGLT2i and GLP-1 RA in patients with T2DM, along with the guidelines that recommend their use, their application in practice remains limited (18–20). Several studies have reported that the rate of using SGLT2i or GLP-1 RA among patients with T2DM and at high risk of or with established ASCVD or CVD ranges from 4.1 to 19% (18, 20, 21). The underutilization of these medications may be attributed to the older age of these patients, as well as the increased number of comorbidities, costs, and formulary barriers (18, 22). Other factors that limit the use of SGLT2i and GLP-1 RA may be related to the prescriber's specialty, concerns about the safety of these medications, or the need for prior authorization (19, 23).

Most previous studies have examined the underutilization of SGLT2i and GLP-1 RA in T2DM patients with established CVD or ASCVD (18, 21, 24). However, there are limited data on the effective prescribing of SGLT2i and GLP-1 RA in patients with T2DM and other compelling indications who are more likely to benefit from using these medications. Thus, this study aimed to describe the prescribing patterns of GLP-1 RA and SGLT2i in patients with T2DM.

## Methods

### Study design and patients

This multicenter retrospective cross-sectional study was conducted in two centers in Riyadh, Saudi Arabia: a secondary care hospital, King Abdullah bin Abdulaziz University Hospital, and a tertiary care center, King Abdulaziz Medical City. We included patients with T2DM who were 18 years of age or older and who had received care in one of the two centers between January 1, 2020, and December 31, 2020. We excluded patients with other types of DM (type 1 DM or gestational DM). This study was conducted in accordance with the Declaration of Helsinki and was approved by the institutional review board at Princess Nourah bint Abdulrahman University (log number: 21–0291) and King Abdullah International Medical Research Center (Ref. #SP20/477/R). The patients' informed consent was not required because all the data were collected from their electronic medical records after de-identification.

According to the ADA patient-centered approach to using SGLT2i or GLP-1 RA for patients with T2DM (25), the patients were split into two major groups. The first group consisted of “patients who were more likely to benefit from SGLT2i or GLP-1 RA” and the second group, “patients who were less likely to benefit from SGLT2i or GLP-1 RA.” The preference criteria for using SGLT2i or GLP-1 RA were based on the ADA recommendations and are detailed in Table 1 (25). Patients who were more likely to benefit from these agents and were prescribed them were categorized as *preferably prescribed*. In contrast, patients who were more likely to benefit from either GLP-1 RA or SGLT-i but were not prescribed these medications were categorized as *underprescribed*. Patients who were less likely to benefit from GLP-1 RA or SGLT2i and were not prescribed these medications were categorized as *preferably not prescribed*, but if they were prescribed them, then they were categorized as *prescribed but unpreferred*.

**Table 1:** The definition of SGLT2i and GLP-1 RA preference criteria based on the ADA recommendations (25).

**Patients' criteria to likely benefit from the use of either SGLT2i or GLP-1 RA**	**SGLT2i for patients with T2DM and one of the following:**
	1–Chronic kidney disease. 2–Established ASCVD. 3–High-risk of ASCVD; defined as having a 10-year ASCVD risk score ≥20%, having stenosis, or left ventricular hypertrophy. 4–BMI ≥ 27 Kg/m^**2**^. 5–HFrEF (< EF <45%). 6–Patient is on two other antidiabetic medications*. **GLP-1 RA for patients with T2DM and one of the following:** 1–Chronic kidney disease. 2–Established ASCVD. 3–High-risk of ASCVD; defined as having a 10-year ASCVD risk score ≥20%, having stenosis, or left ventricular hypertrophy. 4–BMI ≥ 27 Kg/m^**2**^. 5–Patient is on insulin. 6–Patient is on two other antidiabetic medications^*^.

SGLT2i, Sodium–glucose Cotransporter−2 Inhibitors; GLP−1 RA, Glucagon–like Peptide−1 Receptor Agonists; ASCVD, Atherosclerotic Cardiovascular Disease; T2DM, Type 2 diabetes mellitus, BMI, body mass index, HFrEF, Heart failure with reduced ejection fraction; Stenosis, Diagnosis of stenosis of the carotid, coronary, or lower extremity with > 50%. *This is used to indicate the patient has uncontrolled T2DM.

### Data collection and study outcomes

The data were collected from the patients' electronic medical records and included their demographic information and past medical history (PMH) during clinic visits or upon hospital admission. In addition, information about SGLT2i and GLP-1 RA, doses, and prescribers was gathered. The primary outcome was the proportion of patients who were or were not prescribed GLP-1 RA or SGLT2i as the preferred approach according to ADA standards (25). The secondary outcomes were factors associated with the underprescribing of GLP-1 RA or SGLT2i and the distribution of prescriber specialties.

### Statistical analysis

Descriptive statistics, which included means with standard deviations for continuous variables and frequencies with percentages for categorical variables, were used to summarize the study variables. The unpaired ***t***-test for continuous variables and a chi-squared test for categorical variables were used to make an unadjusted comparison between the ***preferably prescribed*** and the ***underprescribed*** groups. Multivariable logistic regression analysis was used to identify the patients' characteristics associated with underprescribing SGLT2i and GLP-1 RA. The data were collected from the patients' electronic medical records using the Research Electronic Data Capture (REDCap^**^®^**^) software, version 7.3.6, and then analyzed using the SAS^**^®^**^ software, version 9.4 (SAS Institute Inc., Cary, NC).

## Results

### Patients' clinical characteristics

The study included 1,220 patients from the two centers. The overall mean age was 59.3 ± 13.2 years, and the majority of the patients were female (56.9%). The most common PMH in our patients was dyslipidemia (69.0%), followed by hypertension (67.6%), as shown in Table 2. According to the ADA patient-centered criteria listed in Table 1; 1,167 patients were categorized as “more likely to benefit from SGLT2i or GLP-1 RA,” while the remaining 53 patients were classified as less likely to benefit from either SGLT2i or GLP-1 RA (Table 2).

**Table 2:** Patients' baseline characteristics (***n*** = 1220).

		**Patients more likely to benefit from the use of either SGLT2i or GLP**−**1 RA**		**Patients less likely to benefit from the use of either SGLT2i or GLP−1 RA**
		**(*****n*** = **1167)**		(*n* = 53)*
**Characteristics**	**Overall**	**Preferably prescribed**	**Underprescribed**	**Preferably not prescribed**
	***n* = 1220**	**(*n* = 222)**	**(*n* = 945)**	**(*n* = 51)**
Age in years	59.3 ± 13.2	57.3 ± 11.4	60.0 ± 13.3	54.1 ± 14.4
Sex, Female	694 (56.9)	139 (62.6)	523 (55.3)	31 (60.8)
BMI (kg/m2)	31.9 ± 6.5	34.2 ± 6.9	31.7 ± 6.3	24.4 ± 2.4
Smoker or former smoker	93 (7.6)	18 (8.1)	70 (7.4)	5 (9.8)
Family history of T2DM	81 (6.8)	13 (5.9)	62 (6.8)	6 (12.2)
**Past medical history**				
Hypertension	825 (67.6)	144 (64.9)	661 (69.9)	19 (37.3)
Dyslipidemia	842 (69.0)	178 (80.2)	634 (67.1)	29 (56.9)
Hypothyroidism	161 (13.2)	36 (16.2)	115 (12.2)	9 (17.6)
Hyperthyroidism	13 (1.1)	2 (0.9)	11 (1.2)	0 (0.0)
Retinopathy	57 (4.7)	25 (11.3)	31 (3.3)	1 (2.0)
Neuropathy	35 (2.9)	14 (6.3)	20 (2.1)	1 (2.0)
**ASCVD 10–year score category**				
Low risk (<5%)	188 (24.1)	56 (33.5)	124 (21.3)	8 (26.7)
Borderline risk (5–7.5%)	103 (13.2)	20 (12.0)	76 (13.1)	6 (20.0)
Intermediate risk (≥7.5– <20%)	250 (32.1)	51 (30.5)	183 (31.5)	16 (53.3)
High risk (≥20%)	238 (30.6)	40 (24.0)	198 (34.1)	0 (0.0)

Data are presented as frequency (%) or mean ± SD. p–values are from the Chi–square test for categorical variables and the t–test for continuous variables.

SGLT2i, Sodium–glucose Cotransporter−2 Inhibitors; GLP−1 RA, Glucagon–like Peptide−1 Receptor Agonists; ASCVD, Atherosclerotic Cardiovascular Disease; T2DM, Type 2 diabetes mellitus; SD, Standard Deviation.

ASCVD includes stroke, transient ischemic attack, acute coronary syndromes (ACSs), a history of myocardial infarction, stable or unstable angina, coronary or other arterial revascularization, or peripheral arterial disease (PAD) presumed to be of atherosclerotic origin.

*Two patients received GLP−1 RA or SGLT2i while not being preferred to be prescribed these medications.

In the patient group, which included those who were less likely to benefit from either SGLT2i or GLP-1 RA, the commonly observed PMHs were dyslipidemia and hypertension (56.9% and 37.3%), respectively. The mean age at 54.1 ± 14.4 years and the mean BMI of 24.4 ± 2.4 kg/m^2^ was much lower than those of the patient group who were more likely to benefit from SGLT2i or GLP-1 RA. In the patients who were less likely to benefit from SGLT2i or GLP-1 RA, more than half had an intermediate risk of ASCVD (53.3%), while 26.7% had a low risk of ASCVD, as shown in Table 2.

The characteristics of the patients who were more likely to benefit from SGLT2i or GLP-1 RA were mostly balanced between patients who received SGLT2i or GLP-1 RA and those who did not, except for the number of female patients and the presence of dyslipidemia, retinopathy, and neuropathy, which were significantly higher among patients who were preferably prescribed SGLT2i or GLP-1 RA, as shown in Table 3. In contrast, the mean age was significantly higher among patients who were underprescribed SGLT2i or GLP-1 RA compared to those prescribed these agents (60.0 ± 13.3 vs. 57.3 ± 11.4 years, *p* = 0.002). In the underprescribed group, significantly more patients had established ASCVD, including stroke/ transient ischemic attack (TIA), compared to the preferably prescribed group (20.7% vs. 14.0%, *p* = 0.0217, 10.6% vs. 4.1%, *p* = 0.0026, respectively; Table 3).

**Table 3:** Factors associated with the under–prescribing of SGLT2i or GLP−1 RA for patients with type 2 diabetes mellitus (*N* = 1167).

**Factors**	**Overall**	**Preferably prescribed**	**Underprescribed**	**Unadjusted analysis**	**Adjusted analysis**
	**(*n* = 1167)**	**(*n* = 222)**	**(*n* = 945)**	***p*–value**	**AOR (95%CI)**
Age in years	59.5 ± 13.0	57.3 ± 11.4	60.0 ± 13.3	0.0020	1.02 (1.01 −1.03)
Sex, female	662 (56.7)	139 (62.6)	523 (55.3)	0.0492	0.78 (0.56 −1.08)
Smoker or former smoker	88 (7.5)	18 (8.1)	70 (7.4)	0.7220	——
Family history of T2DM	75 (6.6)	13 (5.9)	62 (6.8)	0.6613	——
**Past medical history**					
Hypertension	805 (69.0)	144 (64.9)	661 (69.9)	0.1407	——
Dyslipidemia	812 (69.6)	178 (80.2)	634 (67.1)	0.0001	0.45 (0.31 −0.66)
Hypothyroidism	151 (12.9)	36 (16.2)	115 (12.2)	0.1060	——
Hyperthyroidism	13 (1.1)	2 (0.9)	11 (1.2)	0.7368	——
Retinopathy	56 (4.8)	25 (11.3)	31 (3.3)	<0.0001	0.36 (0.19 −0.67)
Neuropathy	34 (2.9)	14 (6.3)	20 (2.1)	0.0008	0.47 (0.21 −1.08)
**Patients' criteria to be prescribed SGLT2i or GLP−1 RA**					
Chronic kidney disease	106 (9.1)	17 (7.7)	89 (9.4)	0.4115	——
HFrEF (EF <45%)	37 (3.2)	7 (3.2)	30 (3.2)	0.9869	——
Established ASCVD	227 (19.5)	31 (14.0)	196 (20.7)	0.0217	——
Myocardial infarction	83 (7.1)	11 (5.0)	72 (7.6)	0.1636	1.86 (0.85 −4.07)
Stable angina	10 (0.9)	1 (0.5)	9 (1.0)	0.4646	——
Unstable angina	18 (1.5)	2 (0.9)	16 (1.7)	0.3879	——
Stroke/TIA	109 (9.3)	9 (4.1)	100 (10.6)	0.0026	2.86 (1.33 −6.15)
PAD	6 (0.5)	0 (0.0)	6 (0.6)	0.2339	——
Revascularization, including stent or CABG	96 (8.2)	18 (8.1)	78 (8.3)	0.9433	0.53 (0.27 −1.04)
**Indicators of high risk**					
ASCVD 10–year risk score ≥20%	238 (20.4)	40 (18.0)	198 (21.0)	0.3288	——
Stenosis	51 (4.4)	9 (4.1)	42 (4.4)	0.7956	——
Left ventricular hypertrophy	68 (5.8)	7 (3.2)	61 (6.5)	0.0588	2.09 (0.91 −4.80)
Obesity (BMI ≥ 27 Kg/m2)	933 (79.9)	190 (85.6)	743 (78.6)	0.0405	0.70 (0.45 −1.09)
**Patient is currently on**					
Two other antidiabetic medications	753 (64.5)	152 (68.5)	601 (63.6)	0.1723	——
Insulin	503 (43.1)	122 (55.0)	381 (40.3)	<0.0001	0.54 (0.40 −0.75)

Data are presented as frequency (%) or mean ± SD. p–values are from the Chi–square test for categorical variables and the t–test for continuous variables.

SGLT2i, Sodium–glucose Cotransporter−2 Inhibitors; GLP−1 RA, Glucagon–like Peptide−1 Receptor Agonists; ASCVD, Atherosclerotic Cardiovascular Disease; T2DM, Type 2 diabetes mellitus; HFrEF, Heart failure with reduced ejection fraction; SD, Standard Deviation; AOR, Adjusted odds ratio.

ASCVD: stroke, transient ischemic attack, acute coronary syndromes (ACSs), a history of myocardial infarction, stable or unstable angina, coronary or other arterial revascularization, or peripheral arterial disease presumed to be of atherosclerotic origin.

### SGLT2i or GLP-1 RA prescribing patterns

Among patients who were more likely to benefit from SGL2i or GLP-1 RA (*n* = 1167), only 19.0% (*n* = 222) received SGLT2i or GLP-1 RA, while the remaining 81.0% did not receive either. In comparison, of the patients who were less likely to benefit from either SGLT2i or GLP-1 RA (*n* = 53), only two received SGLT2i or GLP-1 RA. The main reasons for using SGLT2i or GLP-1 RA in the patients who were preferably prescribed these agents were obesity (BMI ≥ 27 kg/m^2^ and T2DM) at 85.6%, uncontrolled DM indicated by using two or more other antidiabetic agents (68.5%), the presence of high risk for ASCVD (23.9%), and established ASCVD (14%), as presented in Table 4. Moreover, patients with established HF or CKD were 3.2% and 7.7%, respectively, in the preferably prescribed group. In contrast, in the underprescribed group, the most common compelling indications for the use of SGLT2i or GLP-1 RA were obesity (78.6%), uncontrolled DM (63.6%), and a high risk for ASCVD (29.5%), followed by 20.7% having established ASCVD (Table 4). Also, 3.2% and 9.4% of the underprescribed group had HF and CKD, respectively.

**Table 4:** Patient–centered preference criteria for using either SGLT2i or GLP−1 RA (*n* = 1167).

**Patient criteria**	**Overall**	**Preferably prescribed**	**Underprescribed**
	***n* = 1167**	**(*n* = 222)**	**(*n* = 945)**
Chronic kidney disease	106 (9.1)	17 (7.7)	89 (9.4)
HFrEF (EF <45%)	37 (3.2)	7 (3.2)	30 (3.2)
Established ASCVD	227 (19.5)	31 (14.0)	196 (20.7)
Myocardial infarction	83 (7.1)	11 (5.0)	72 (7.6)
Stable angina	10 (0.9)	1 (0.5)	9 (1.0)
Unstable angina	18 (1.5)	2 (0.9)	16 (1.7)
Stroke/TIA	109 (9.3)	9 (4.1)	100 (10.6)
PAD	6 (0.5)	0 (0.0)	6 (0.6)
Revascularization, including stent or CABG	96 (8.2)	18 (8.1)	78 (8.3)
Indicators of high risk	332 (28.4)	53 (23.9)	279 (29.5)
ASCVD 10–year risk score ≥20%	238 (20.4)	40 (18.0)	198 (21.0)
Stenosis	51 (4.4)	9 (4.1)	42 (4.4)
Left ventricular hypertrophy	68 (5.8)	7 (3.2)	61 (6.5)
Obesity (BMI ≥ 27 Kg/m^2^)	933 (79.9)	190 (85.6)	743 (78.6)
**Patient is currently on**			
Two other antidiabetic medications	753 (64.5)	152 (68.5)	601 (63.6)
Insulin	503 (43.1)	122 (55.0)	381 (40.3)

Data are presented as frequency (%). p–values are from the Chi–square test.

SGLT2i, Sodium–glucose Cotransporter−2 Inhibitors; GLP−1 RA, Glucagon–like Peptide−1 Receptor Agonists; HFrEF, Heart failure with reduced ejection fraction; ASCVD, Atherosclerotic Cardiovascular Disease; TIA, transient ischemic attack; PAD, peripheral arterial disease presumed to be of atherosclerotic origin; CABG, Coronary artery bypass graft; BMI, Body mass index; Stenosis, Diagnosis of stenosis of the carotid, coronary, or lower extremity with > 50%.

In the patients who received SGLT2i or GLP-1 RA for preferred reasons, 58.2% were prescribed SGLT2i, and 51.8% were prescribed GLP-1 RA. It is worth noting that 22 patients received both SGLT2i and GLP-1 RA. Dapagliflozin (90.7%) and liraglutide (71.4%) were the most commonly prescribed SGLT2i and GLP-1 RA, respectively (Table 5). In addition, the most commonly prescribed antidiabetic medication, along with SGLT2i or GLP-1 RA, was metformin (86%), followed by insulin (55%). The additional antidiabetic agents prescribed with SGLT2i or GLP-1 RA are detailed in Table 5.

**Table 5:** Antidiabetic medication prescribed for patients with type 2 diabetes mellitus (*N* = 1167).

**Antidiabetic medication or class of medications**	**Overall**	**Preferably prescribed**	**Underprescribed**
	**(*n* = 1167)**	**(*n* = 222)**	**(*n* = 945)**
**SGLT2i**	129 (11.1)	129 (58.1)	——
Dapagliflozin	117 (90.7)	117 (52.7)	——
Empagliflozin	12 (9.3)	12 (5.4)	——
**GLP−1 RA**	115 (9.8)	115 (51.8)	——
Liraglutide	81 (70.4)	81 (36.5)	——
Semglutide	33 (28.7)	33 (14.9)	——
Dulaglutide	1 (0.9)	1 (0.4)	——
**Additional antidiabetic agents**			
Metformin	982 (84.1)	191 (86.0)	791 (83.7)
Insulin	503 (43.1)	122 (55.0)	381 (40.3)
DPP−4 inhibitors	438 (37.5)	74 (33.3)	364 (38.5)
Sulphonylurea	381 (32.6)	67 (30.2)	314 (33.2)
Thiazolidinedione	19 (1.6)	6 (2.7)	13 (1.4)

Data are presented as frequency (%).

SGLT2i, Sodium–glucose Cotransporter−2 Inhibitors; GLP−1 RA, Glucagon–like Peptide−1 Receptor Agonists; DPP−4, Dipeptidyl peptidase−4.

### Factors associated with underprescribing

Older age and a history of stroke/TIA were significantly associated with higher odds of underprescribing SGLT2i or GLP-1 RA (OR 1.02; 95% CI 1.01–1.03 and OR 2.86; 95% CI 1.33–6.15, respectively). Conversely, patients with a PMH of dyslipidemia (OR 0.45; 95% CI 0.31–0.66) or retinopathy (OR 0.36; 95% CI 0.19–0.67) had lower odds of being underprescribed either SGLT2i or GLP-1 RA. Fortunately, the odds of underprescribing were significantly lower among patients with T2DM who were receiving insulin (OR 0.54; 95% CI 0.40–0.75). The details of the logistic regression used to assess these factors are presented in Table 3.

### Prescriber specialties

**Figure 1:**
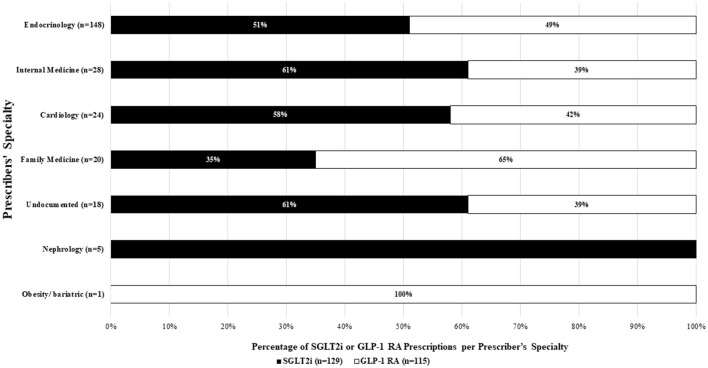
SGLT2i or GLP-1 RA prescribers' specialty (*N* = 224).

Endocrinologists were the most frequent specialty prescribing SGLT2i or GLP-1 RA (60.6%), followed by internal medicine (IM) physicians (11.4%). Cardiologists were responsible for only 9.8% of the SGLT2i or GLP-1 RA prescriptions, and nephrology for 2%. Most of the SGLT2i agents (*n* = 129) were prescribed by endocrinologists (59%), followed by IM physicians (13%) and cardiologists (10.9%). In contrast, GLP-1 RA prescriptions (*n* = 115) were also mostly prescribed by endocrinologists (62.6%), family medicine physicians (11.3%) and then IM physicians (9.6%) accounted for the other significant percentages, as depicted in Figure 1.

## Discussion

This retrospective cross-sectional study evaluated the prescribing patterns of SGLT2i and GLP-1 RA in patients with T2DM. Among the patients who were more likely to benefit from SGLT2i or GLP-1 RA, only 19% were preferably prescribed them for diabetes management or cardioprotective, renoprotective, or weight-reduction benefits. In contrast, 96% of the patients who were less likely to benefit from SGLT2i or GLP-1 RA were not prescribed these agents.

Although SGLT2i or GLP-1 RA are preferred over other antidiabetic medications for their organ protection effect in patients with T2DM (26), we still observed a low rate of prescribing SGLT2i or GLP-1 RA in patients with T2DM and established ASCVD, HF, CKD, or obesity who were likely to benefit from these medications. This low prescribing rate is similar to that found in a previous retrospective cross-sectional study that used a large set of U.S. claims data (27). That study reported that SGLT2i or GLP-1 RA was prescribed in patients with T2DM at a rate of <12% and at an even lower rate in patients with ASCVD (<9%) (27). It is worth noting that the data, in this case, were collected in 2015 before the first set of ADA guidelines (2017) was published, which recommended the use of SGLT2i or GLP-1 RA in patients with T2DM and ASCVD (22, 27). However, a 2020 nationwide study from U.S. Veterans Affairs, which included 537,980 patients with ASCVD and T2DM, found that the prescription of SGLT2i or GLP-1 RA remained low at 19% (21). Similarly, we found that 86% of the patients with established ASCVD and 83% of those at high risk of ASCVD (10-year score ≥ 20%) were not prescribed SGLT2i or GLP-1 RA.

In this study, we noticed a high rate (81%) of underprescribing SGLT2i or GLP-1 RA among patients who were likelier to benefit from them. Specifically, patients with increased age and a history of stroke/TIA had higher odds of being underprescribed SGLT2i or GLP-1 RA. This finding is consistent with a retrospective study that found that the mean age of patients who were not prescribed SGLT2i or GLP-1 RA was greater than that of those who had been prescribed these medications (73.0 ± 9 years vs. 69.2 ± 8 years, *p* < 0.01 for SGLT2i; 72.8 ± 9 vs. 69.6 ± 8, *p* < 0.01 for GLP-1 RA). Moreover, they found a significantly greater proportion of patients with ischemic cerebrovascular disease among the nonusers of GLP-1 RA and SGLT2i compared to the users (27.4% vs. 23.9%, *p* < 0.01 for SGLT2i; 27.2% vs. 25.6%, *p* < 0.01 for GLP-1 RA) (21), which is similar to what we found in our study.

The underprescription of GLP-1 RA and SGL-2i could be attributed to patient-related factors, such as a patient's preference to avoid using multiple medications for diabetes management, or the claim that they would comply with a healthy diet and regular exercise to avoid additional agents. Moreover, in the present study, the underprescribing of SGLT2i or GLP-1 RA in older patients may be attributed to lenient glycemic control goals. In addition, there is some concern about increased comorbidities, a high risk of polypharmacy and side effects such hypoglycemia specially in older adult patients. All of these reasons may prevent prescribers from initiating the use of additional agents. Another interesting observation in this study was that 91% of the patients with a history of stroke/TIA were not prescribed SGLT2i or GLP-1 RA, despite the recommendation to use these medications in patients with established ASCVD, including cerebrovascular diseases (9). This underutilization in patients with stroke/TIA may have been influenced by the fact that most of the available evidence supports the use of SGLT2i or GLP-1 RA for primary prevention. Still, limited studies have proven their benefits for secondary stroke prevention in patients with DM (8–31).

The results also revealed that the odds of underprescribing GLP-1 RA and SGLT2i were significantly lower in patients with a history of dyslipidemia, retinopathy, or on insulin therapy. A retrospective cohort study of patients with T2DM and CVD or CV risk factors (e.g., hyperlipidemia, hypertension, obesity, smoking, or micro/macroalbuminuria) found high rates of prescribing SGLT2i (OR 3.94; 95% CI 3.90–3.99) or GLP-1 RA (OR 1.19; 95% CI 1.17–1.20) in patients at risk of CV (20). In that study, the likelihood of using SGLT2i or GLP-1 RA increased with obesity or dyslipidemia diagnosis, as in our study (20). Since high lipid levels are the main driver of ASCVD, this could be suggested as a marker for prescribers to initiate SGLT2i or GLP-1 RA in patients with established ASCVD who are at high risk for ASCVD, or even obese patients. The use of insulin therapy in patients with T2DM may indicate uncontrolled DM, since it is recommended to start with hemoglobin A1C levels above 10% (9). Insulin therapy may also be combined with GLP-1 RA to exhibit longer glycemic durability (9). However, the fear of hypoglycemia resulting from the combination of insulin with SGLT2i or GLP-1 RA may lead to the underprescription of these agents in patients using insulin.

Furthermore, this underutilization of SGLT2i and GLP-1 RA may be driven by prescriber or payor factors, such as high costs, limited prescriber awareness, and formulary restrictions (22, 31). Insurance companies may also deny coverage of these drugs and push for alternative agents (18, 22). In the present study, most of the SGLT2i and GLP-1 RA prescriptions were given by endocrinologists (60.6%), followed by IM physicians (11.4%). The prescription rates of SGLT2i and GLP-1 RA by cardiologists and nephrologists were low. At the same time, 84% of the patients with CKD, 86% with established ASCVD, and 81% with HF were not prescribed SGLT2i or GLP-1 RA. This may indicate the limited prescribers' knowledge about the effective use and the cardiovascular and renal benefits of SGLT2i and GLP-1 RA in patients with T2DM and these comorbidities. The trends in the specializations of the prescribers are similar to national and global models, where endocrinologists and primary care physicians are the most common prescribers for SGLT2i and GLP-1 RA, while cardiologists are the least common prescribers (32, 33). A cross-sectional survey in Saudi Arabia that included 103 prescribers showed that 15.5% of prescribers admitted to choosing SGLT2i as a second-line therapy, whereas 31.1% chose it as a first-line therapy for patients with CVD (34).

The behavior of underprescribing SGLT2i or GLP-1 RA is consistent with the findings of previous studies that have highlighted the issue of physicians' lack of familiarity with the effective utilization of these agents (23, 33). Since SGLT2i and GLP-1 RA are relatively new agents, providers may be reluctant to prescribe them (21, 23). Cardiologists and nephrologists, in particular, tend to be hesitant to prescribe SGLT2i or GLP-1 RA, as they view the use of antidiabetic medications to be beyond their scope (35). Moreover, they may be concerned about the adverse effects of SGLT2i, such as volume status and other safety concerns, especially in patients with CVD or CKD. The limited prescription of SGLT2i or GLP-1 RA may also be influenced by institutional limitations on the scope of SGLT2i and GLP-1 RA (21) Prescribers, including cardiologists and nephrologists, need to understand that the decision to use SGLT2i or GLP-1 RA is based on their cardiorenal protection, independent of these medications' hypoglycemic effects. SGLT2i and GLP-1 RA are currently recommended in patients, regardless of the presence of T2DM (13, 15, 16). More importantly, the latest ADA standards recommend SGLT2i or GLP-1 RA as first-line therapy in patients with an established or high risk of ASCVD, HF, or CKD (10, 14). These new recommendations emphasize the importance of prescribers assessing the advantages of using these agents in patients who would likely benefit from them, regardless of DM control.

Our study is one of several that have assessed the real-world prescribing patterns of SGLT2i and GLP-1 RA in patients with T2DM (18, 20, 21, 27). Most previous studies have focused on the rate of prescribing SGLT2i or GLP-1 RA in patients with established CVD or ASCVD (18, 21, 27). However, this study assessed all patients with T2DM, regardless of their history or risk for ASCVD, to determine if SGLT2i or GLP-1 RA are prescribed according to a personalized approach and individual comorbidities. However, the study has several limitations, such as its retrospective design and small sample size, which may limit the generalizability of its results. Specifically, the number of patients prescribed SGLT2i or GLP-1 RA was relatively small, which may have limited our ability to identify additional factors that might have affected the prescribing of these medications. Also, we cannot exclude the possibility that the use of SGLT2i or GLP-1 RA may be driven by the medications' hypersensitivity, drug-to-drug interaction, or failing other agents, which were not investigated in the present study.

## Conclusions

This study concurs with previous studies highlighting the underutilization of GLP-1 RA and SGLT2i in patients with T2DM, but this time in patients with T2DM and additional compelling indications. To optimize the use of GLP-1 RA and SGLT2i for their additional benefits, prescribers need to follow the most recent guidelines in managing patients with T2DM. Thus, increasing prescribers' awareness about the appropriate use of GLP-1 RA and SGLT2i is essential. In light of the expected increases in the use of GLP-1 RA and SGLT2i with the new recommendations, larger-scale studies are needed to investigate their effective use.

## Data availability statement

The original contributions presented in the study are included in the article/supplementary material, further inquiries can be directed to the corresponding author.

## Ethics statement

This study was conducted following the Declaration of Helsinki and was approved by the Princess Nourah bint Abdulrahman University (PNU) Institutional Review Board (IRB) (IRB Log Number: 21–0291) and King Abdullah International Medical Research Center (Ref.#333 SP20/477/R).

## Author contributions

All authors listed have made a substantial, direct, and intellectual contribution to the work and approved it for publication.

## Funding

This work was supported by Princess Nourah bint Abdulrahman University Researchers Supporting Project number (PNURSP2022R78), Princess Nourah bint Abdulrahman University, Riyadh, Saudi Arabia.

## Conflict of interest

The authors declare that the research was conducted in the absence of any commercial or financial relationships that could be construed as a potential conflict of interest.

## Publisher's note

All claims expressed in this article are solely those of the authors and do not necessarily represent those of their affiliated organizations, or those of the publisher, the editors and the reviewers. Any product that may be evaluated in this article, or claim that may be made by its manufacturer, is not guaranteed or endorsed by the publisher.

## Abbreviations

DM: Diabetes mellitus
SA: Saudi Arabia
ASCVD: atherosclerotic cardiovascular disease
T2DM: type two diabetes mellitus
SGLT2i: sodium-glucose cotransporter-2 inhibitors
GLP-1 RA: glucagon-like peptide-1 receptor agonists
CHD: coronary heart disease
PAD: peripheral artery disease
HF: heart failure
MACE: major adverse cardiac events
CV: cardiovascular
CVD: Cardiovascular disease
BMI: body mass index
PMH: past medical history
HFrEF: heart failure with a reduced ejection fraction
TIA: transient ischemic attack
IM: internal medicine
ADA: American Diabetes Association.

## References

[1] Zinman B Wanner C Lachin JM Fitchett D Bluhmki E Hantel S. Empagliflozin, cardiovascular outcomes, and mortality in type 2 diabetes. N Engl J Med. (2015) 373:2117–28. 10.1056/NEJMoa1504720 26378978

[2] Perkovic V Jardine MJ Neal B Bompoint S Heerspink HJL Charytan DM. Canagliflozin and renal outcomes in type 2 diabetes and nephropathy. N Engl J Med. (2019) 380:2295–306. 10.1056/NEJMoa1811744 30990260

[3] Wiviott SD Raz I Bonaca MP Mosenzon O Kato ET Cahn A. Dapagliflozin and cardiovascular outcomes in type 2 diabetes. N Engl J Med. (2018) 380:347–57. 10.1056/NEJMoa1812389 30415602

[4] McMurray JJv Solomon SD Inzucchi SE Køber L Kosiborod MN Martinez FA. Dapagliflozin in patients with heart failure and reduced ejection fraction. N Engl J Med. (2019) 381:1995–2008. 10.1056/NEJMoa1911303 31535829

[5] Packer M Anker SD Butler J Filippatos G Pocock SJ Carson P. Cardiovascular and renal outcomes with empagliflozin in heart failure. N Engl J Med. (2020) 383:1413–24. 10.1056/NEJMoa2022190 32865377

[6] Marso SP Daniels GH Brown-Frandsen K Kristensen P Mann JFE Nauck MA. Liraglutide and cardiovascular outcomes in type 2 diabetes. N Engl J Med. (2016) 375:311–22. 10.1056/NEJMoa1603827 27295427 PMC4985288

[7] Marso SP Bain SC Consoli A Eliaschewitz FG Jódar E Leiter LA. Semaglutide and cardiovascular outcomes in patients with type 2 diabetes. N Engl J Med. (2016) 375:1834–44. 10.1056/NEJMoa1607141 27633186

[8] Gerstein HC Colhoun HM Dagenais GR Diaz R Lakshmanan M Pais P. Dulaglutide and cardiovascular outcomes in type 2 diabetes (REWIND): a double-blind, randomised placebo-controlled trial. Lancet. (2019) 394:121–30. 10.1016/S0140-6736(19)31149-3 31189511

[9] American Diabetes Association Professional Practice Committee. 9. Pharmacologic approaches to glycemic treatment: standards of medical care in diabetes-−2022. Diabetes Care. (2022) 45:S125–43. 10.2337/dc22-S009 34964831

[10] Davies MJ Aroda VR Collins BS Gabbay RA Green J Maruthur NM. Management of hyperglycemia in type 2 diabetes, 2022. A consensus report by the American Diabetes Association (ADA) and the European Association for the Study of Diabetes (EASD). Diabetes Care. (2022) 23:dci220034. 10.2337/dci22-0034 36148880 PMC10008140

[11] Committee ADAPP. 16. Diabetes care in the hospital: standards of medical care in diabetes-−2022. Diabetes Care. (2021) 45:S244–53. 10.2337/dc22-S016 34964884

[12] Cosentino F Grant PJ Aboyans V Bailey CJ Ceriello A Delgado V. 2019 ESC Guidelines on diabetes, pre-diabetes, and cardiovascular diseases developed in collaboration with the EASD. Eur Heart J. (2020) 41:255–323. 10.1093/eurheartj/ehz486 31497854

[13] Kidney Disease: Improving Global Outcomes (KDIGO) Diabetes Work Group. KDIGO 2020 clinical practice guideline for diabetes management in chronic kidney disease. J Int Soc Nephol. (2020) 98:S1–115. 10.1016/j.kint.2020.06.019 32998798

[14] de Boer IH Khunti K Sadusky T Tuttle KR Neumiller JJ Rhee CM. Diabetes Management in chronic kidney disease: a consensus report by the American Diabetes Association (ADA) and Kidney Disease: Improving Global Outcomes (KDIGO). Diabetes Care. (2022) 3:dci220027. 10.2337/dci22-0027 36189689 PMC9870667

[15] McDonagh TA Metra M Adamo M Gardner RS Baumbach A Böhm M. 2021 ESC Guidelines for the diagnosis and treatment of acute and chronic heart failure. Eur Heart J. (2021) 42:3599–726. 10.1093/eurheartj/ehab368 34447992

[16] Heidenreich PA Bozkurt B Aguilar D Allen LA Byun JJ Colvin MM. 2022 AHA/ACC/HFSA Guideline for the Management of Heart Failure: a report of the American College of Cardiology/American Heart Association Joint Committee on Clinical Practice Guidelines. Circulation. (2022) 145:1073. 10.1161/CIR.0000000000001073 35363499

[17] American Diabetes Association Professional Practice Committee. 8. Obesity and weight management for the prevention and treatment of type 2 diabetes: standards of medical care in diabetes-−2022. Diabetes Care. (2022) 45:S113–24. 10.2337/dc22-S008 34964843

[18] Pantalone KM Misra-Hebert AD Hobbs TM Ji X Kong SX Milinovich A. Antidiabetic treatment patterns and specialty care utilization among patients with type 2 diabetes and cardiovascular disease. Cardiovasc Diabetol. (2018) 17:54. 10.1186/s12933-018-0699-7 29636104 PMC5892008

[19] Khunti K Jabbour S Cos X Mudaliar S Mende C Bonaca M. Sodium-glucose co-transporter-2 inhibitors in patients with type 2 diabetes: Barriers and solutions for improving uptake in routine clinical practice. Diabetes Obes Metab. (2022) 24:1187–96. 10.1111/dom.14684 35238129 PMC9313799

[20] Mody R Meyers J Yu M Davis K Levine JA. Are we there yet? Increasing use of cardioprotective antihyperglycemic agents in patients with T2D and CVD or CV risk in the United States. Curr Med Res Opin Taylor & Francis. (2022) 27:1–11. 10.1080/03007995.2022.2085962 35758147

[21] Mahtta D Ramsey DJ Lee MT Chen L. al Rifai M, Akeroyd JM, et al. Utilization rates of SGLT2 inhibitors and GLP-1 receptor agonists and their facility-level variation among patients with atherosclerotic cardiovascular disease and type 2 diabetes: insights from the department of veterans affairs. Diabetes Care. (2022) 45:372–80. 10.2337/dc21-1815 35015080 PMC8914426

[22] Eberly LA Yang L Eneanya ND Essien U Julien H Nathan AS. Association of race/ethnicity, gender, and socioeconomic status with sodium-glucose cotransporter 2 inhibitor use among patients with diabetes in the US. JAMA Netw Open. (2021) 4:e216139. 10.1001/jamanetworkopen.2021.6139 33856475 PMC8050743

[23] Gao Y Peterson E Pagidipati N. Barriers to prescribing glucose-lowering therapies with cardiometabolic benefits. Am Heart J. (2020) 224:47–53. 10.1016/j.ahj.2020.03.017 32304879

[24] Adhikari R Jha K Dardari Z Heyward J Blumenthal RS Eckel RH. National Trends in Use of sodium-glucose cotransporter-2 inhibitors and glucagon-like peptide-1 receptor agonists by cardiologists and other specialties, 2015 to 2020. J Am Heart Assoc. (2022) 11:e023811. 10.1161/JAHA.121.023811 35475341 PMC9238581

[25] American Diabetes Association. Pharmacologic approaches to glycemic treatment: standards of medical care in diabetes—2019. Diabetes Care. (2019) 42:S90–102. 10.2337/dc19-S009 30559235

[26] American Diabetes Association Professional Practice Committee. 10. Cardiovascular disease and risk management: standards of medical care in diabetes—2022. Diabetes Care. (2022) 45:S144–74. 10.2337/dc22-S010 34964815

[27] Weng W Tian Y Kong S Ganguly R Hersløv M Brett J. The prevalence of cardiovascular disease and antidiabetes treatment characteristics among a large type 2 diabetes population in the United States. Endocrinol Diabetes Metab. (2019) 2:e00076. 10.1002/edm2.76 31294089 PMC6613222

[28] Wiviott SD Raz I Bonaca MP Mosenzon O Kato ET Cahn A. Dapagliflozin and cardiovascular outcomes in type 2 diabetes. N Engl J Med. (2019) 380:347–57. 30415602 10.1056/NEJMoa1812389

[29] Cannon CP Pratley R Dagogo-Jack S Mancuso J Huyck S Masiukiewicz U. Cardiovascular outcomes with ertugliflozin in type 2 diabetes. N Engl J Med. (2020) 383:1425–35. 10.1056/NEJMoa2004967 32966714

[30] Chen R Ovbiagele B Feng W. Diabetes and Stroke: Epidemiology, pathophysiology, pharmaceuticals and outcomes. Am J Med Sci. (2016) 351:380–6. 10.1016/j.amjms.2016.01.011 27079344 PMC5298897

[31] Luo J Feldman R Rothenberger SD Hernandez I Gellad WF. Coverage, formulary restrictions, and out-of-pocket costs for sodium-glucose cotransporter 2 inhibitors and glucagon-like peptide 1 receptor agonists in the medicare part D program. JAMA Netw Open Am Med Assoc. (2020) 3:e2020969–e2020969. 10.1001/jamanetworkopen.2020.20969 33057641 PMC7563069

[32] Somaili M Oraibi O Mohrag M Hommadi A Moafa E Kulaybi A. Baseline characteristics associated with sodium-glucose cotransporter inhibitor prescriptions in type 2 diabetic patients in Jazan, Saudi Arabia. Cureus Cureus. (2022) 14:e24284–e24284. 10.7759/cureus.24284 35602773 PMC9119415

[33] Muthiah V Vasanth S Avinainder S. P MC, Arman Q, L JJ, et al. Prescriber patterns of SGLT2i after expansions of US food and drug administration labeling. J Am Coll Cardiol. (2018) 72:3370–2. 10.1016/j.jacc.2018.08.2202 30409566

[34] Allyhiani M Kurdi A Abdulaziz A Faqeh S Alhajjaji A Alansari S. Prescribing patterns of antidiabetics in type 2 diabetes and factors affecting them. Saudi Pharm J. (2021) 30:112–9. 10.1016/j.jsps.2021.12.019 35528857 PMC9072689

[35] Slater TA Drozd M Palin V Bowles C Waduud MA Khatib R. Prescribing diabetes medication for cardiovascular risk reduction in patients admitted with acute coronary syndromes: a survey of cardiologists' attitudes and practice. Eur Heart J. (2020) 6:194–6. 10.1093/ehjcvp/pvz058 31702003 PMC7225869

